# Expression of leukemia inhibitory factor (LIF) and its receptor gp190 in human liver and in cultured human liver myofibroblasts. Cloning of new isoforms of LIF mRNA

**DOI:** 10.1186/1476-5926-3-10

**Published:** 2004-11-26

**Authors:** Toru Hisaka, Alexis Desmoulière, Jean-Luc Taupin, Sophie Daburon, Véronique Neaud, Nathalie Senant, Jean-Frédéric Blanc, Jean-François Moreau, Jean Rosenbaum

**Affiliations:** 1 INSERM, E362, Bordeaux, F-33076 France; Université Victor Segalen Bordeaux 2, Bordeaux, F-33076 France; 2 CNRS, UMR 5164, Bordeaux, F-33076 France; Université Victor Segalen Bordeaux 2, Bordeaux, F-33076 France; 3 IFR 66, 33076 Bordeaux France; 4 Kurume University School of Medicine, Department of Pathology, Fukuoka, Japan

## Abstract

**Background:**

The cytokine leukemia inhibitory factor (LIF) mediates its biological effects through binding to its high affinity receptor made of the low-affinity LIF receptor subunit gp190 (LIF-R) and the gp130 subunit. LIF exerts several important effects in the liver, however, data on liver expression of LIF are scarce. The aim of this study was to examine the expression of LIF and LIF-R in human liver.

**Results:**

LIF expression, analyzed by immunohistochemistry, was barely detectable in normal liver but was strong within cirrhotic fibrous septa and was found in spindle-shaped cells compatible with myofibroblasts. Accordingly, cultured human liver myofibroblasts expressed high levels of LIF as shown by ELISA and Northern blot. Biological assay demonstrated that myofibroblast-derived LIF was fully active. RT-PCR showed expression of the LIF-D and M isoforms, and also of low levels of new variants of LIF-D and LIF-M resulting from deletion of exon 2 through alternative splicing. LIF receptor expression was detected mainly as a continuous sinusoidal staining that was enhanced in cirrhotic liver, suggestive of endothelial cell and/or hepatocyte labeling. Immunohistochemistry, flow cytometry and STAT-3 phosphorylation assays did not provide evidence for LIF receptor expression by myofibroblasts themselves. LIF secretion by cultured myofibroblasts was down regulated by the addition of interleukin-4.

**Conclusions:**

We show for the first time the expression of LIF in human liver myofibroblasts, as well as of two new isoforms of LIF mRNA. Expression of LIF by myofibroblasts and of its receptor by adjacent cells suggests a potential LIF paracrine loop in human liver that may play a role in the regulation of intra-hepatic inflammation.

## Background

Leukemia inhibitory factor (LIF) belongs to the interleukin (IL)-6 family of cytokines, together with IL-11, ciliary neurotrophic factor, cardiotrophin-1, oncostatin M and neurotrophin-1/B cell stimulating factor-3. LIF is widely expressed in tissues and in many isolated cells. LIF expression is commonly up-regulated during inflammation. Nevertheless, its role seems to be complex as both pro- and anti-inflammatory properties have been described for that cytokine. Although LIF, like IL-6, is able to drive a significant acute-phase reaction in non-human primates [1], this has been questioned in humans [2]. LIF exerts its biological activities through its binding to a hetero-oligomeric receptor complex between the low-affinity LIF receptor subunit gp190 and the signal-transducing subunit gp130. The gp130 subunit is common to all members of the IL-6 family.

Several isoforms of LIF consecutive to alternative splicing have been described. The second and third exons are common to all isoforms, whereas there are 3 alternate first exons – D, M, and T. The fate of the mature LIF molecule is highly dependent on exon 1 usage; thus, the human LIF-D transcript encodes a secreted protein that is biologically active and can signalize via the LIF receptor. The human LIF-M transcript does not contain any in-frame AUG, but it is known to be translated into both secreted and intracellular proteins [3]. The secreted LIF-M protein can also be found sequestered in the extracellular matrix where it is biologically active [4]. Finally, the first exon from the human LIF-T, which does not contain any in-frame AUG, is responsible for the synthesis of an intracellular protein with a leucine zipper motif that might function as a transcription factor [5].

As outlined above, LIF is potentially involved in liver physiology and pathophysiology; however, data on liver expression of LIF are scarce. LIF expression was not detected in normal rat liver but it was highly induced following partial hepatectomy, mainly in non- parenchymal cells [6], suggesting its involvement in liver regeneration. To our knowledge, the expression of LIF has not been described in human liver.

Therefore, the aim of this study was to examine the expression of LIF and of its specific receptor gp190 in human liver. Results obtained with immunostaining of liver sections led us to examine LIF expression by cultured liver myofibroblasts, cells that play a major role in liver fibrogenesis.

## Results

### LIF expression

**Figure 1:**
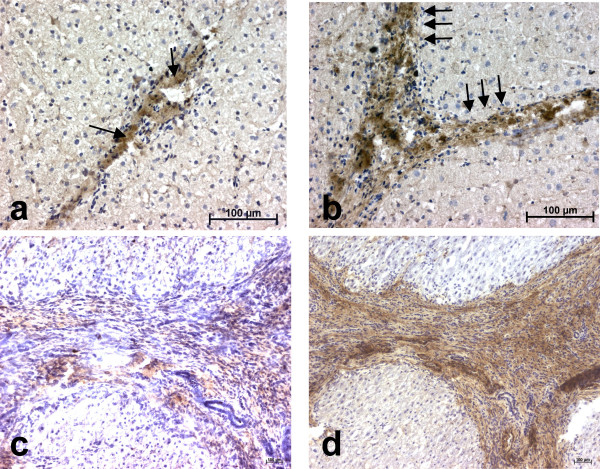
**Immunohistochemical analysis of LIF expression in normal and cirrhotic human liver. **(**a**): LIF expression is seen in normal liver in the stroma of portal tracts (arrows); (**b**): LIF is strongly expressed in fibrotic septa in cirrhotic liver (arrows); (**c**) and (**d**): consecutive sections of a cirrhotic liver analyzed for LIF (**c**) or alpha-smooth muscle actin (**d**) expression. No labeling was seen when the antibodies were replaced by a species-matched control antibody.

Human liver tissues were examined for LIF expression by immunohistochemistry. In normal liver, a faint but consistent LIF expression was detected in the stroma of portal tracts (Fig. 1A). No signal was observed along sinusoids. In fibrotic liver tissues, an intense expression of LIF was seen along fibrous septa which is consistent with the presence of myofibroblasts (Fig. 1B). Staining adjacent sections with LIF antibody and with an antibody to alpha-smooth muscle actin (that labels myofibroblasts) suggested a large degree of colocalization (Figs. 1C,1D). Part of the LIF staining also appeared to be extracellular. There was no difference in the type of staining whatever the etiology of liver fibrosis. No labeling was found when the LIF antibody was replaced by a species-matched control antibody.

**Figure 2:**
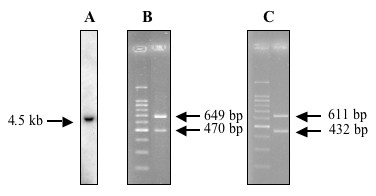
**Detection of LIF transcripts in cultured human liver myofibroblasts. **(**A**): Northern blot. Total RNA from cultured human liver myofibroblasts was hybridized with a cDNA probe to human LIF. A single 4.5 kb band was observed; (**B**) and (**C**): RT-PCR. Total RNA was subjected to reverse transcription then to PCR with the hLIF-D3/hLIF-N4 (**B**) or with the hLIF-M3/hLIF-N4 primers (**C**).

**Figure 3:**
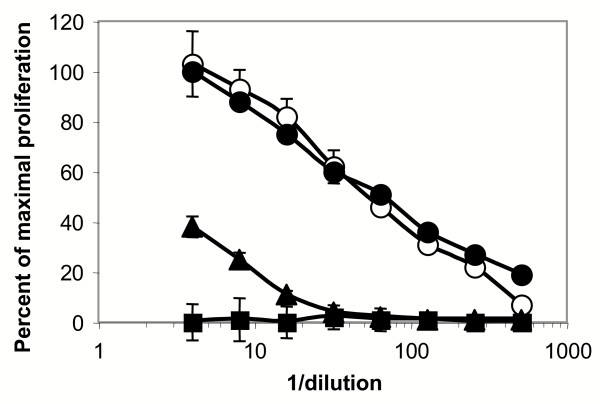
**Biological activity of myofibroblast-derived LIF. **BaF3 cells stably transfected with the gp130 and the gp190 subunits were exposed to dilutions of recombinant human LIF (starting concentration: 4 ng/ml) (open circles), culture medium (filled squares), myofibroblast conditioned medium alone (filled circles) or together with the blocking anti-gp190 antibody 12D3 at 20 μg/ml (filled triangles). Cell growth was monitored with a colorimetric assay. The figure shows the mean ± SD of 3 experiments performed in duplicate (SD are not always visible due to their small size).

Analysis of total RNA from cultured human liver myofibroblasts by Northern blot revealed a single 4.5 kb transcript (Fig. 2A). RT-PCR experiments, described in more detail later, demonstrated the expression of both D and M isoforms of LIF (Fig. 2B). When cell supernatants were tested with an ELISA assay specific for human LIF, levels ranged between 800 and 8 000 ng/ml in different isolates. In order to make sure that this corresponded to biologically active LIF, the supernatants were tested for their ability to promote the growth of the LIF-dependent cell line BaF3, stably transfected with the human gp190 and gp130 isoforms. As shown in Fig. 3, myofibroblasts supernatants efficiently stimulated the growth of these cells in a dose-dependent fashion, confirming that biologically active LIF was effectively produced. Furthermore, the effect on BaF3 transfectants growth was abolished in the presence of the blocking gp190 LIF receptor antibody 12D3. The results of the ELISA combined with the 100 fold inhibition of biological activity, seen after anti-gp190 addition, further confirmed that most of the BaF3 growth-promoting activity produced by cultured myofibroblasts is likely to be LIF.

**Figure 4:**
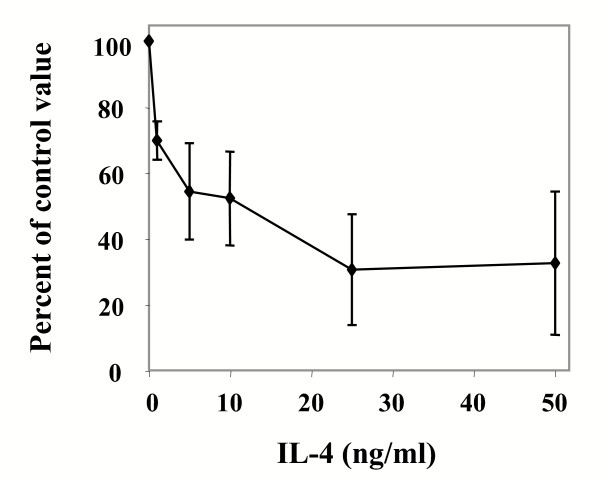
**Regulation of LIF secretion by interleukin-4. **Confluent cultures of human liver myofibroblasts were cultured in the presence of the indicated concentrations of IL-4, for 48 h in serum-free medium. LIF was measured by ELISA in the supernatant and the results were normalized according to the DNA content of the monolayer (mean ± SD of 3 experiments). The effect of IL-4 was highly significant, as assessed by ANOVA (p = 0.001).

As shown in Figure 4, when cells were incubated with graduated amount of recombinant human IL-4, the constitutive LIF secretion was dose-dependently reduced, demonstrating that this production may be regulated *in vivo*.

### Cloning of new isoforms of LIF mRNA

**Figure 5:**
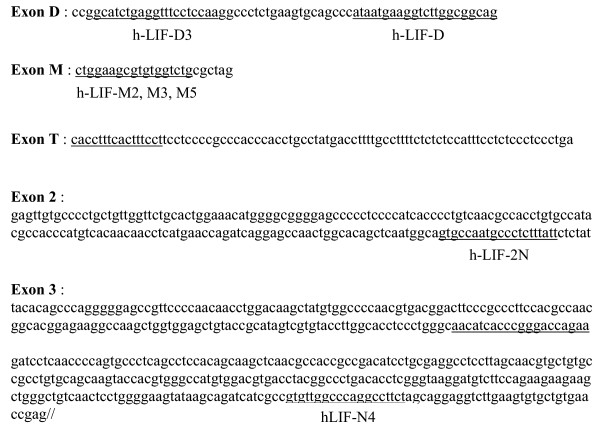
**Sequence of LIF-D, M and T isoforms. **Exons D, M and T are the 3 alternate first exons. Primers used for PCR are underlined. Primers hLIF-M2, M3 and M5 cover the same sequence but differ because of the presence or the absence of restriction sites.

In order to test whether myofibroblasts transcribed all the alternatively spliced D, M or T first exons, a first set of RT-PCR experiments was carried out using the forward primers chosen in the alternative D, M or T first exons (hLIF-D3, hLIF-M3 and hLIF-T5), and a common reverse primer chosen in exon 3 (hLIF-N4) (Table 1 and Fig. 5). As shown in Fig. 2B, PCR with D- or M-specific primers was positive. Moreover, it always yielded a second, shorter, PCR product in addition to the expected amplified product (Fig. 2B). Similar results were obtained with other primer sets specific for either LIF-D (hLIF-D) or LIF-M (h-LIFM5) combined with hLIF-3N (data not shown), which strengthened the previous observation. No amplification products were obtained with the T primer. Then, we designed a reverse primer within exon 2 (hLIF-2N) that was used in conjunction with the forward hLIF-D and hLIF-M2 primers. In that case, we detected only a product of the expected size for both D and M PCRs (not shown).

**Table 1:** Primers used for PCR

**Primer**	Sequence (*): 5'> 3'	**Orientation**	**Ref.**
hLIF-D	ATAATGAAGGTCTTGGCGGCAG	Forward	
HLIF-D3	AAA*CTGCAG*GCATCTGAGGTTTCCTCCAA	Forward	
hLIF-M2	CTGGAAGCGTGTGGTCTG	Forward	
HLIF-M3	AAA*CTGCAG*CTGGAAGCGTGTGGTCTG	Forward	
hLIF-M5	TA*GAATTC*TGGAAGCGTGTGGTG	Forward	[3]
hLIF-T5	AT*GAATTC*TGTCACCTTTCACTTTCCT	Forward	[3]
hLIF-2N	AATAAAGAGGGCATTGGCAC	Reverse	
hLIF-3N	TTCTGGTCCCGGGTGATGTT	Reverse	[3]
HLIF-N4	GC*TCTAGA*GAAGGCCTGGGCCAACAC	Reverse	

(*) Bases in italics refer to restriction sites.

The sizes of the additional products obtained with the hLIF-N4 primer were shorter by about 200 bp, which is the exact size of exon 2, raising therefore the possibility that the shorter PCR products were derived from a hitherto not described mRNA species where exon 2 was deleted through alternative splicing. In order to investigate this possibility, the short D and M fragments were cloned into a plasmid and sequenced. Sequencing indeed revealed that the first exon (either D or M) was directly spliced to the third one resulting in new, short transcripts that we have designated s-LIF-D and s-LIF-M.

**Figure 6:**
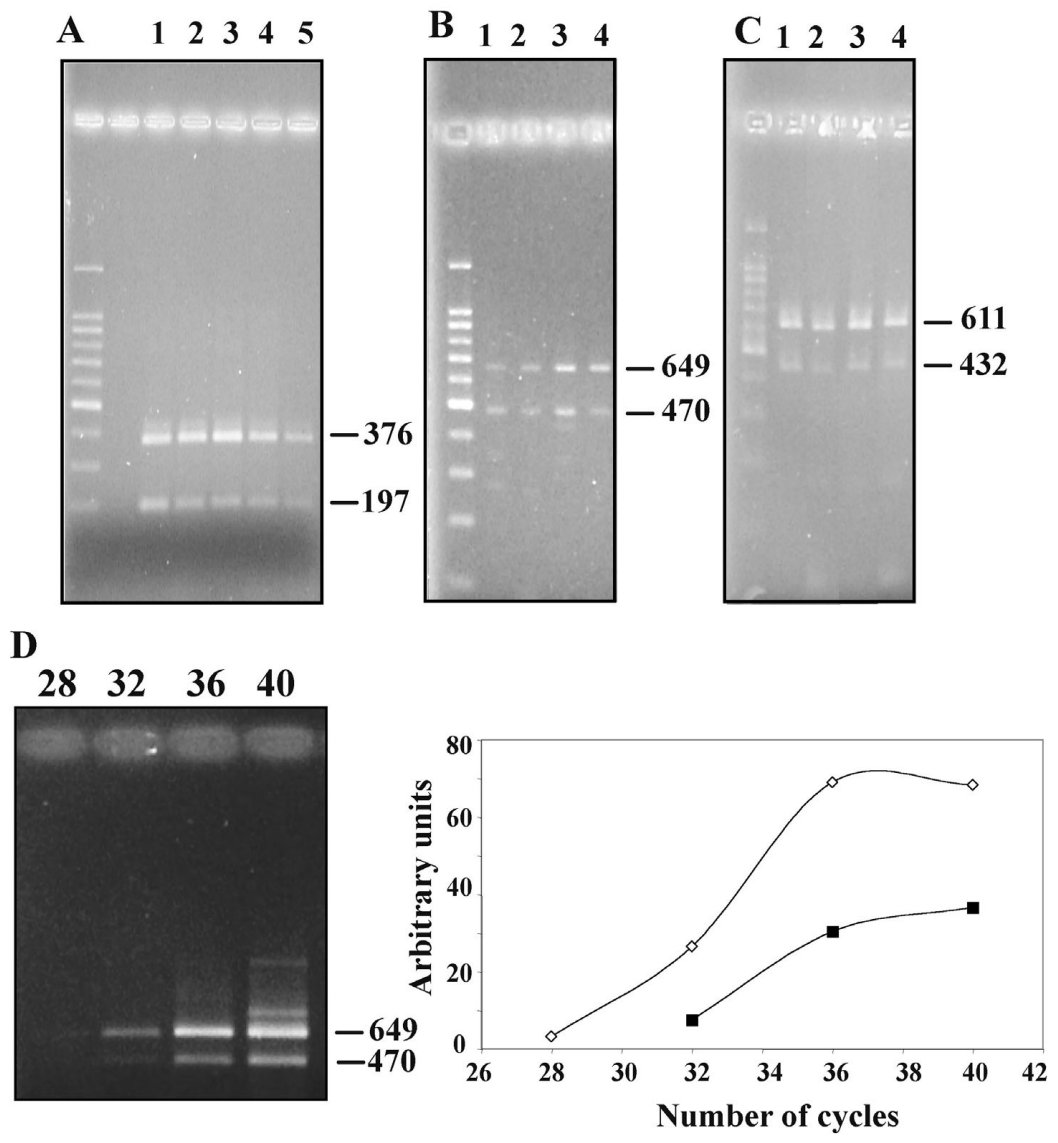
**RT-PCR analysis of LIF-M expression in various cell lines and in human liver. **(**A**): LIF-M expression was analyzed with the hLIF-M2 and hLIF-3N primers: Line 1, human liver myofibroblasts; Line 2, HepG2; Line 3, Hep3B; Line 4, HuH7; Line 5, HEK293. Product sizes are shown in bp; (**B**): normal human liver samples. LIF-D expression was analyzed with the hLIF-D3 and hLIF-N4 primers in 4 different samples. The same samples also expressed LIF-M (not shown). Product sizes are shown in bp; (**C**): diseased human liver samples. In that case, LIF-M expression was analyzed with the hLIF-M3 and hLIF-4N primers in 4 cases of cirrhotic liver. The same samples also expressed LIF-D (not shown). Product sizes are shown in bp; (**D**): semi-quantitation of LIF-D and s-LIF-D expression in a human liver myofibroblasts sample. LIF-D expression was analyzed with the hLIF-D3 and hLIF-N4 primers. The left part shows the migration pattern of the PCR-amplified products with the number of cycles above and the size of the products indicated by arrows, in bp. The graph on the right shows the signal quantification. Similar results were obtained with LIF-M.

The existence of these alternate transcripts could be observed in several hepatocellular carcinoma cell lines (HepG2, HuH7, Hep3B) and in the HEK293 cell line, derived from embryonic human kidney (Fig. 6A). They were also expressed in normal human liver samples (Fig. 6B) as well as in cirrhotic ones (Fig. 6C).

The relative abundance of the variant transcripts relative to the classical transcripts was studied using a semi-quantitative RT-PCR method, where PCR was carried out for varying cycles numbers. As can be seen in Fig. 6D, expression of the s-LIF-D transcript lagged several cycles behind that of the long transcript. Similar results were obtained with the s-LIF-M transcript (not shown).

### LIF receptor expression

**Figure 7:**
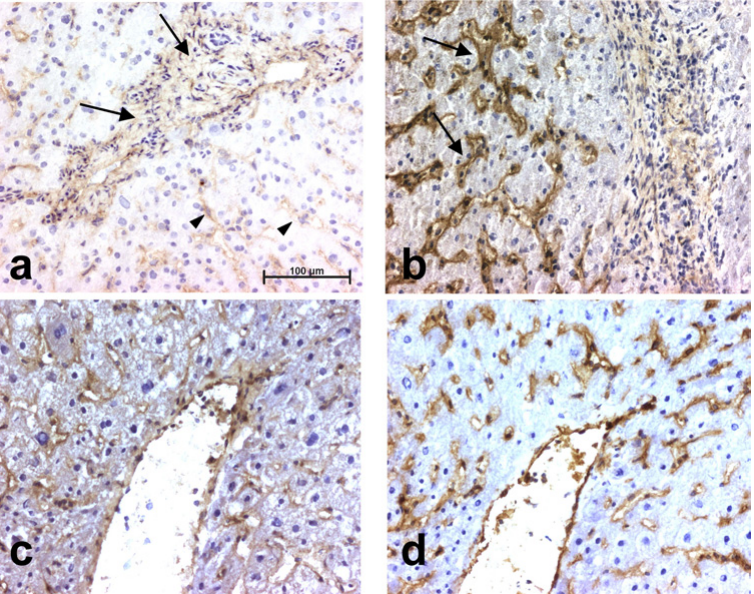
**Detection of LIF receptor by immunohistochemistry. **(**a**): LIF-R expression in normal liver is observed in portal tracts (arrows) as well as along sinusoids (arrowheads); (**b**): Sinusoidal staining is highly increased in cirrhotic liver (arrows); (**c**) and (**d**): consecutive sections of a cirrhotic liver analyzed for LIF-R (**c**) or CD31 (**d**) expression. No labeling was seen when the antibodies were replaced by a species-matched control antibody.

The expression of the gp190 subunit by liver cells was then examined by immunohistochemistry. Five different antibodies, directed against separate epitopes, were used and yielded similar results. In normal liver tissue, LIF receptor (LIF-R) expression was detected as a continuous sinusoidal staining and in the stroma of portal tracts (Fig. 7A). In the cirrhotic liver, the sinusoidal staining was enhanced, whereas a very faint staining was observed in fibrous septa (Fig. 7B). Staining adjacent sections with LIF receptor antibody and with an antibody to CD31 (endothelial cells in the cirrhotic liver were labeled) showed a large degree of colocalization (Figs 7C,7D).

In a subsequent step, cultured human liver myofibroblasts were examined for their membrane expression of gp190 using flow cytometry. Adherent cells were released by action of EDTA and subjected to anti-gp190 labeling. No detectable levels of gp190 were observed with any of the 5 antibodies, although gp130 expression could be detected with the B-R3 antibody. In order to detect a low-level expression of functional LIF-R, myofibroblasts were exposed for 15 minutes to 10 ng/ml recombinant LIF; then, STAT-3 phosphorylation was examined by Western blot. No consistent effects were seen in 7 separate experiments. When a very weak signal was occasionally seen, it was not inhibited by 2 separate blocking antibodies to LIF-R (data not shown). Finally, production of soluble receptor was never detected in myofibroblast supernatants either.

## Discussion

In this study, we demonstrate for the first time that LIF is expressed at low levels in normal human liver, whereas it is greatly increased in fibrotic liver, in a localization consistent with that of activated myofibroblasts. The slightly diffuse staining is suggestive of extracellular matrix deposition consistent with the expression of the M-type isoform of LIF. Experiments using cultured human liver myofibroblasts confirmed that these cells secreted extremely high levels of LIF in the range of 0.1–1 μg/10^6 ^cells/48 h. These levels are similar to those produced by activated lymphocytes, a classic source of LIF, and suggest that liver myofibroblasts may be a major source of LIF during chronic liver diseases. Our results are in agreement with data obtained in the rat showing that non-parenchymal cells, possibly activated stellate cells (*i.e.*, myofibroblasts), express LIF [6]. Another study also reported an increased expression of LIF in peri-ductular cells, following bile duct ligation in IL-6 knock-out mice [7]; this location likely qualifies those cells as myofibroblasts. LIF expression by liver myofibroblasts is also reminiscent of its expression by kidney mesangial cells, a close relative to liver myofibroblasts, that we have previously reported [8].

On the other hand, and in contrast with mesangial cells [9], liver myofibroblasts do not appear to express cell surface LIF-specific gp190 receptor subunit. This is based on results obtained from immunohistochemistry, flow cytometry, as well as functional experiments. This indicates that LIF cannot exert an autocrine effect on liver myofibroblasts. However, we show that myofibroblasts express the IL-6 family common transducing subunit gp130. In this regard, others have shown that human liver myofibroblasts are responsive to oncostatin-M [10], indicating the presence of its functional alternative receptor consisting of gp130 and the specific OSMRβ chain.

Nonetheless, LIF receptor expression was detected by immunohistochemistry in human liver, in a peri-sinusoidal location. Similar results were obtained with 5 different antibodies directed to several epitopes of gp190. The pattern of continuous sinusoidal staining and the colocalization experiments are in favor of an expression in sinusoidal endothelial cells. However, we can not exclude staining of the sinusoidal domain of hepatocytes. In any case, these data indicate that cells close to LIF-producing myofibroblasts express LIF receptors and could thus respond to LIF in a paracrine fashion.

This study led to the discovery of new LIF transcripts resulting from a direct splicing of exon 1 to exon 3. This was observed for both LIF-D and LIF-M. Those transcripts were present at much lower levels than full-length transcripts, as suggested by RT-PCR and by the fact that they do not appear on Northern blot; thus, their biological relevance can be questioned. Whether s-LIF-D or s-LIF-M transcripts are translated also remains hypothetical. In the case of s-LIF-D, initiation at the AUG within exon D would result in a reading-frame shift following the 6^th ^amino-acid (aa) and a termination at aa 88, the resulting protein bearing no homology with LIF. There are, however, several in-frame CUG codons within exon 3. Initiation at CUG 113 would result in the synthesis of a 125 aa polypeptide, recapitulating the sequence of the C-terminal part of LIF. Similar considerations apply to s-LIF-M that, in any case, does not contain an initiating AUG in exon 1. It should be emphasized that the lack of an AUG codon does not preclude the translation of the classical forms of LIF-M or LIF-T [3,5]. More experiments are needed to know whether these new transcripts are translated.

LIF secretion was dose-dependently decreased by IL-4, a known inhibitor of LIF secretion in other cell types [11,12]. IL-4 is also known to up-regulate collagen synthesis in human liver myofibroblasts and could thus be a pro-fibrogenic mediator in the liver [13]. Whether LIF expression is relevant to liver fibrogenesis needs to be assessed. LIF could affect extracellular matrix remodeling since it regulates the expression of several matrix proteinases and their inhibitors in various cell types [14,15]. In addition, LIF could play a role in the pathophysiology of chronic liver diseases through action on endothelial cells and on hepatocytes. Regarding endothelial cells, and depending on the model, both pro-angiogenic [16] and anti-angiogenic effects [17] have been described. Especially interesting is the demonstration that LIF can stimulate the adhesion of neutrophils to endothelial cells [18]; indeed, neutrophils are involved in the pathogenesis of liver diseases such as alcoholic liver disease. As already mentioned, the effects of LIF on human hepatocytes are still being debated [2].

## Conclusions

For the first time, we show the expression of LIF in human liver myofibroblasts, as well as of two new isoforms of mRNA. Hepatic stellate cells and activated myofibroblasts have already been shown to synthesize a number of mediators involved in the control of inflammation, such as monocyte chemotactic-1 protein [19], or platelet-activating factor [20]. Expression of LIF by myofibroblasts and of its receptor by adjacent cells suggest a potential LIF paracrine loop in human liver that may play a role in the regulation of intra-hepatic inflammation and reinforces the concept of a major role of liver myofibroblasts in the regulation of intra-hepatic inflammation [21].

## Methods

### Tissue samples

Histologically normal/subnormal liver samples were obtained from macroscopically normal location in hepatectomy specimens, taken at a distance from a focal nodular hyperplasia (n = 5); a hemangioma (n = 1); or a colon cancer metastasis (n = 1). Cirrhotic specimens (n = 11) were obtained from patients undergoing liver transplantation for cirrhosis with associated hepatocellular carcinoma. In 10 out of 11 cases, the patients underwent liver transplantation. The cirrhosis etiologies were viral hepatitis C (n = 4); viral hepatitis B + D (n = 2); alcoholic (n = 4); or a combination of viral hepatitis B + C + alcoholic (n = 1).

### Tissue sampling and processing

A portion of fresh tissue samples was routinely formalin-fixed and paraffin-embedded for diagnosis and a portion immediately frozen in liquid nitrogen-cooled isopentane and stored at -80°C. Five μm-thick serial frozen sections of each sample were air-dried on Super Frost/Plus slides (Menzel Glaser, Germany) and processed for immunohistochemistry. The procedures were in accordance with the European guidelines for the use of human tissues.

### Materials

Culture medium and additives, recombinant human epidermal growth factor (EGF) and Moloney Murine Leukemia Virus reverse transcriptase were from Gibco-BRL (Life Technologies, Cergy-Pontoise, France). Taq polymerase and the pGEM-Teasy plasmid were from Promega (Madison, WI). The Qiagen RNeasy minikit was from Qiagen (Courtaboeuf, France). The [α^32^P]dCTP, Hybond N^+ ^membrane, ECL reagent, and the Ready-to-go DNA labeling kit were from Amersham (Les Ulis, France). Ultrahyb solution was from Ambion (Austin, TX). Recombinant human IL-4 was a gift from Schering-Plough (Kenilworth, NJ). Anti-gp130 mAb B-R3 was from Diaclone (Besançon, France), anti-gp80 mAb M91 was from Coulter-Immunotech (Marseille, France), anti-phospho-STAT-3 (Tyr705) was from Cell Signaling Technology (Beverly, MA). All other chemicals were from Sigma (St Quentin Fallavier, France).

### Cell culture

Human hepatic myofibroblasts were obtained from explants of non-tumoral liver resected during partial hepatectomy and characterized as previously described [22,23]. Myofibroblasts were routinely grown in DMEM containing 5% fetal calf serum, 5% pooled human AB serum and 5 ng/ml EGF. For studies of LIF secretion, cells were grown to confluence, made quiescent in serum and EGF-free Waymouth medium for 2 days and then exposed to agonists for 2 days. The results were normalized according to the DNA content of the monolayer [24].

### Detection of LIF in culture supernatants

#### ELISA

Human LIF was measured using an ELISA based on two specific monoclonal antibodies, exactly as described previously [25]. A standard curve was obtained with recombinant glycosylated CHO-derived human LIF. The detection limit of the assay is 20 pg/ml, and LIF can be quantified at concentrations up to 1.2 ng/ml, without sample dilution. This ELISA is not sensitive to soluble receptor binding to the LIF molecule.

#### Biological assay

The Ba/F3 proliferation assays were performed, as described previously [26], using the Ba/F3 gp190 + gp130 transfectant cell line which expresses the two human LIF receptor chains (gp190 and gp130) and responds to all cytokines sharing gp190. LIF-dependent Ba/F3 cells were washed three times with RPMI to remove LIF which is required to maintain the cell line; then, cells (5 × 10^3 ^per well, in 50 μl, in duplicates) were incubated in the presence of 50 μl of three-fold dilutions of cytokines or supernatant, as indicated. After three days at 37°C, 0.015 ml of a 5 mg/ml solution of 3-(4,5-dimethylthiazol-2-yl)-2,5-diphenyl tetrazolium bromide (Sigma, Saint-Quentin Fallavier, France), in PBS, was added to each well. After 4 hours at 37°C, 0.11 ml of a mixture of 95 % isopropanol + 5 % formic acid was added to the wells, and the absorbance values were read at 570 nm, in a Titertek Multiskan microplate reader (Labsystems, Les Ullis, France). The blank consisted of eight wells containing the cells incubated with the Ba/F3 culture medium without any added cytokine.

### Detection of LIF mRNA by Northern blot

Total RNA was isolated using the Qiagen RNeasy minikit. For Northern blot, 2 μg RNA were separated on a 1.0% agarose gel containing ethidium bromide in MOPS buffer. Running buffer and gel contained 0.2 M formaldehyde. The RNAs were transferred onto a Hybond N^+ ^membrane by downward capillary transfer in running buffer. Examination of the stained membrane under UV light was used to confirm the quality of loading and transfer. The probe used was a 730 bp cDNA containing the whole coding sequence of human LIF [27]. Probes were labeled with [α^32^P]dCTP, by random priming using the Ready-to-go kit. Hybridization was performed using the Ultrahyb solution. The blots were washed in stringent conditions (0.1X SSC, 0.1% SDS at 65°C).

### RT-PCR and cloning

One μg of total RNA was reverse-transcribed using MMLV-RT. An aliquot was used for PCR. Thirty five cycles were performed, each consisting of 94°C, 30 s; 60°C, 30 s; and 72°C, 30 s. PCR was performed in 50 μl of a reaction buffer containing 50 mM KCl, 10 mM Tris-HCl (pH 9.0), 1% Triton X-100, 1.5 mM MgCl_2_, 0.4 mM dNTPs, 0.2 mM primers, and 1.25 units of Taq polymerase. Then, an aliquot of the reaction was analyzed by agarose gel electrophoresis. The primers used are listed in Table 1 and are also positioned on the LIF sequence in Figure 5. When indicated, PCR products were directly cloned in the pGEM-Teasy plasmid and sequenced on both strands (Genome Express, Meylan, France).

### Detection of LIF and LIF receptor expression

#### Antibodies and immunoperoxidase histochemistry

A commercially available polyclonal antibody against human LIF (R&D Systems, Minneapolis, Minnesota, USA), and different monoclonal antibodies against gp190, previously described [28], were used at concentrations optimised on control tissues. For colocalization experiments, mouse monoclonal antibodies against α-smooth muscle actin (Dako A/S, Glostrup, Denmark), and CD 31 (Dako) were used. For immunohistochemistry, frozen sections were incubated with the antibodies diluted in phosphate-buffered saline, pH 7.4, containing 4% bovine serum albumin. After washing, the epitopes were detected with the Envision^+ ^system HRP detection and revealed with liquid diaminobenzidine (Dako). As negative control, we used either a clarified mouse myeloma ascites (Cappel Research Products, Durham, USA) or a rabbit non-immune immunoglobulin fraction (Dako), at the same concentration as the respective antibodies. Sections were examined with a Zeiss Axioplan 2 microscope (Carl Zeiss Microscopy, Jena, Germany). Images were acquired with an AxioCam camera (Carl Zeiss Vision, Hallbergmoos, Germany) by means of the AxioVision image processing and analysis system (Carl Zeiss Vision).

#### Flow cytometry

For each staining, 2 × 10^5 ^cells were incubated for 30 min at 4°C with saturating concentrations (10 μg/ml) of the indicated antibody in 0.1 ml of PBS supplemented with 1 % bovine serum albumin (BSA) and 0.1 % human polyclonal IgG (w/v, both from Sigma). Then, cells were washed twice with the same buffer and incubated for 30 min at 4°C with the FITC-conjugated goat anti-mouse IgG. After washing with PBS, the cells were resuspended in 0.14 ml of PBS containing 1% formaldehyde (v/v) and analysed by flow cytometry with a three color FACScalibur flow cytometer (Becton-Dickinson, Mountain View, CA) equipped with the CellQuest software. Control stainings used the second antibody only.

#### ELISA (soluble receptor)

The sandwich ELISA assay for soluble gp190 measurement has already been described [28]. It uses mAb 6G8 as the capture mAb, and biotinylated 10B2 mAb as the tracing mAb. Both mAb recognize distinct epitopes specific to the ectodomain of gp190. The assay has a detection limit of 0.5 ng/ml.

### Immunodetection of phosphorylated STAT-3

Confluent cultures of myofibroblasts were left for 2 days in serum-free medium, and subsequently exposed to recombinant human LIF for 15 minutes [29]. Then, cells were lyzed in modified RIPA buffer in the presence of protease and phosphatase inhibitors, as described [30]. Identical amounts of proteins were analyzed by Western blot with an antibody against phospho-STAT-3. The blots were stripped and rehybridized with an antibody against total STAT-3.

## Authors' contributions

TH performed most of the cell culture and RT-PCR experiments and cloned the new LIF variants. AD and NS performed the immunohistochemistry experiments and prepared the corresponding figures. JLT provided the monoclonal antibodies to LIF-R and participated in the design of the experiments showing the secretion of active LIF. SD performed the LIF ELISA assays, the biological activity testing and flow cytometry experiments. VN performed the experiments looking at STAT-3 phosphorylation. JFB provided the human liver samples. JFM was involved in the coordination of the project and in the critical reading of the manuscript. JR conceived the study and was the main coordinator and responsible for the redaction of the manuscript. All authors read and approved the final manuscript.
